# Supplementary material for: Bi‐allelic VPS16 variants limit HOPS/CORVET levels and cause a mucopolysaccharidosis‐like disease
Source: EMBO Mol Med. 2021 May 3;13(5):e13376. doi: 10.15252/emmm.202013376 (PMC8103096; doi:10.15252/emmm.202013376)
Supplement: Supplementary file 1 — Appendix [file EMMM-13-e13376-s003.pdf]

Bi-allelic VPS16 variants limit HOPS/CORVET levels and cause a mucopolysaccharidosis-like disease – APPENDIX

Table of contents

Extended patient description……………………………………………………...page 2

Supplementary references……………………………………………………...page 5

Appendix figure S1 (related to Fig 6C-D)...……………………………………...page 6

Appendix table S1 (related to Fig 3-8, EV3-EV4)..……………………………..page 7

EXTENDED PATIENT DESCRIPTIONS

Patient A is a boy born at 34 weeks of gestation to healthy first-cousin parents of Iranian descent (Family I; Fig. 2A). He was born via acute caesarean section due to vaginal bleeding (underlying cause is unknown) with a birth weight of 2.5 kg [z-score +0.87 (Chou et al. 2020)], length of 50 cm (z +3.37), and head circumference of 33 cm (z +0.53). He was healthy and developed normally until the age of 9 months, when the first signs of psychomotor regression appeared following a viral gastroenteritis. He could sit with aid but no longer crawled, and from then on failed to attain new psychomotor skills. A first brain CT was normal. An X-ray of the hand performed at 10 months of age was interpreted as normal.

From 14 months of age, the boy displayed periodic neutropenia and progressively developed muscular hypotonia and lower limb spasticity. At the age of 1 year and 9 months he was referred for further etiological investigations. Clinical examination revealed global developmental delay corresponding to a developmental age of 6 months, central hypotonia with severe head lag during traction, lower limb dystonia, impaired neck and shoulder mobility and coarse facial features suggestive of a mucopolysaccharidosis (Fig. 1A). An MRI of the brain showed abnormal myelination and a thin corpus callosum. EEG revealed generalized encephalopathy, but no seizure activity was observed. Neurophysiological testing showed signs of demyelinating sensorymotor polyneuropathy. Cerebrospinal fluid (CSF) biomarkers were extremely high (tau protein 27400 ng/L, ref <250 ng/L; neurofilament light 8970 ng/L, ref <380 ng/L; glial fibrillary acidic protein 2950 ng/L, ref <250 ng/L; albumin 2020 ng/L, ref <225 ng/L).

Urine glycosaminoglycans, plasma amino acids, acylcarnitine profile and urine organic acids were normal. Extended screening for LSDs showed increased excretion of total sialic acid in urine (142 mmol/mol creatinine, ref 50–107 mmol/mol creatinine) and an abnormal pattern of both acidic and neutral oligosaccharides. Enzymatic assays for aspartylglucosaminidase (AGU), α-*N*-acetylgalactosaminidase (Schindler), α-fucosidase (α-fucosidosis) and hexosaminidase A/B (GM_2-gangliosidosis) failed to find the cause for these abnormalities. Metachromatic leukodystrophy, Krabbe, Gaucher, Wolman and Mucolipidosis II/III were also excluded by enzymatic measurements. Levels of globotriaosylsphingosine (lysoGb3) and glucosylsphingosine were normal. Chitotriosidase was slightly elevated (61 nkat/L, ref <40 nkat/L). Biochemical assays for peroxisomal diseases (pipecolic, pristanic and phytanic acids, very long chain fatty acids and plasmalogenes) were normal. Vacuolized lymphocytes in blood were increased (8%, ref <4%), and a bone marrow smear revealed densely stained granules in myelopoetic cells (Fig. 1I), suggestive of an LSD (e.g. Chediak-Higashi syndrome). An NGS-based exome panel targeting known LSD genes did not reveal any pathogenic variants. The patient was diagnosed with severe obstructive sleep apnea, due to adenoid and tonsil hypertrophy and underwent adeno- and tonsillectomy at the age of 1 year and 11 months with moderate improvement of sleep quality. The patient was perceived to have normal visual

function despite slow tracking eye movements, while fundoscopic examination revealed mild pallor of the optic discs suggestive of optic atrophy.

Follow-up MRI of brain and spinal cord at 2 years and 4 months revealed patchy white matter lesions in the periventricular and deep white matter (Fig. 1E). A decreased N-acetylaspartate (NAA) peak was seen on MR spectroscopy (Fig. 1F). Cerebellar volumes were reduced, suggesting atrophy. Spine MRI showed contrast enhancement of the nerve roots of the conus medullaris (Fig. 1H). MRI of the abdomen was normal.

At last follow-up at age of 3.5 years, the patient showed more prominent dysmorphism with coarse facies, macroglossia and hypertrichosis, as well as thoracic deformity (Fig.1A). His length was 86 cm (z -4), weight 10 kg (z -4.2), body mass index 13.5 kg/m² and head circumference 48.5 cm (z -2.5). His psychomotor development had regressed further to a developmental age of approximately 2 months. He had developed slight knee joint contractures and received botulinum toxin injections and oral baclofen due to lower limb spasticity. Renewed analysis of urine glycosaminoglycans showed increased excretion (21 g/mol creatinine, ref 3–13 g/mol creatinine) and an abnormal pattern (65% chondroitin sulphate, 25% dermatan sulphate and 10% heparan sulphate). An X-ray showed coxa valga. Audiologic tests were inconclusive due to repeated ear infections. He suffered from severe gastroesophageal reflux (despite treatment with protein pump inhibitors), requiring exclusive feeding via a gastrostomy tube. Repeated heart examinations have been normal. Blood analyses have recurrently shown anemia (Hb 92-125 g/L, ref 115-135 g/L), but platelet counts have been normal (151-470×10^9/L, ref 150-350×10^9/L). Neutrophil counts have persisted at or below the lower normal range (absolute neutrophil counts 0.3-4.3×10^9/L, ref 1.5-8.5×10^9/L). He has received oral iron supplementation due to microcytic anemia since the age of 1 year and 10 months. Antibiotic prophylaxis has been given and serious bacterial infections have been prevented.

<u>Patient B</u> is a girl born at 38+4 weeks of gestation after an uneventful pregnancy, to Turkish consanguineous, first-cousin parents (Family II; Fig. 2A), of which the mother had been diagnosed with autoimmune thrombocytopenia but the father reported healthy. Her birth weight was 3.4 kg (z-score +0.36 (WHO Multicentre Growth Reference Study Group 2006), length 51 cm (z +0.99) and head circumference 35 cm (z +0.95). Newborn screening indicated biotinidase deficiency (OMIM #253260), which was confirmed by enzymatic and molecular genetic analyses. Oral biotin supplementation was started immediately. In addition, blood counts showed thrombocytopenia (platelet count 29×10^9/L, ref 84–478×10^9/L). This was interpreted as of alloimmune origin because of the mother's underlying condition, and recovered spontaneously. The patient had recurrent infections requiring antibiotic treatments, beginning on the second postnatal day. During infections, but also at asymptomatic encounters, mild to severe neutropenia was seen (absolute neutrophil counts 0.2-1.5×10^9/L, ref >1.5×10^9/L), while other white blood cells generally showed normal levels. Detection of anti-neutrophil antibodies raised the suspicion for primary

autoimmune neutropenia. Occasionally, neutrophil counts could be restored by administration of granulocyte colony stimulating factor (G-CSF). Furthermore, a normo- to microcytic anemia was repeatedly apparent, even after the period of physiologic anemia of infancy (Hb 74-110 g/L, ref >110 g/L; hematocrit 22.1-33.8 %, ref >33 %; MCV 69.9-80 µM³, ref >70 µM³; reference values for 7–17 months of age according to (Kliegman, 2016). No laboratory indicators for iron deficiency were noted (soluble transferrin receptor was not assessed).

Psychomotor development was normal until 5 months of life, but reduced movements and vocalizations as well as feeding difficulties were noted at 7 months of age. At nine months, she could reach out, grasp, and make hand-to-mouth contact, but not turn around or crawl. Persisting newborn reflexes were seen, including the Galant reaction and the palmomental reflex. An MRI of the brain at this age showed global decreased brain volume with corpus callosum hypoplasia (not shown).

Progressive feeding difficulties and recurrent postprandial vomiting necessitated gastric tube feeding from 15 months of age. At 16 months, pyramidal signs, a striatal toe and severe muscular hypotonia were noted. A follow-up MRI at this age showed lack of progress of myelination (Fig. 1E), in comparison with previous MRI at 9 months of age. Dysmorphic stigmata with coarse facial features, a broad nasal bridge, and bushy eyebrows were obvious (Fig. 1B); both hands appeared broad and the phalanges shortened (Fig. 1B). Hepatomegaly was evident clinically and upon abdominal ultrasound. Markedly elevated levels of CSF protein (7138 mg/L, ref 0.09–0.33 mg/L) and slightly elevated CSF lactate (2.1 mmol/L, ref 1.0–2.0 mmol/L) were seen. CSF levels of 5-methyltetrahydrofolate (5-MTHF) were reduced (20.4 nmol/L, ref 64–182 nmol/L) and neopterin increased (99 nmol/L, ref 7–32 nmol/L).

Enzymatic assays for known LSDs (MPSs I, IIIA-C, IVB and VII, Sandhoff, Tay-Sachs, α- and β-mannosidose, fucosidose and Krabbe's disease), VLCFA, urine organic acid, amino acids in CSF and plasma and a urine mucopolysaccharide screening test were normal or showed unspecific patterns. At 21 months of age, the patient showed lack of fixation and could not vocalize or communicate. No reaching, grasping or voluntary movements could be observed. X-rays of chest, skull, and hand revealed dysostosis multiplex (Fig. 1C-D). An EEG was normal and there was no history of epileptic seizures.

Latest available growth parameter were gathered at 1 year and 5 months: weight 7.6 kg (z-score -2.4), length 74 cm (z -2.1), body mass index 13.9 kg/m² (z -1.5) and head circumference 46 cm (z -0.1). At an age of 2 years and 3 months, the patient died following an episode of acute worsening and fever (details are unavailable).

SUPPLEMENTARY REFERENCES

Chou, J.H., Roumiantsev, S. & Singh, R., 2020. PediTools Electronic Growth Chart Calculators: Applications in Clinical Care, Research, and Quality Improvement. *Journal of Medical Internet Research*, 22(1), pp.e16204–17.

Kliegman, R. M., Stanton, B., St. Geme, J., Schor, N. (2016) *Nelson's textbook of pediatrics (20th edition),* Elsevier, Philadelphia.

WHO Multicentre Growth Reference Study Group (2006) Assessment of differences in linear growth among populations in the WHO Multicentre Growth Reference Study. *Acta paediatrica (Oslo, Norway). Supplement*, 450, pp.56–65.

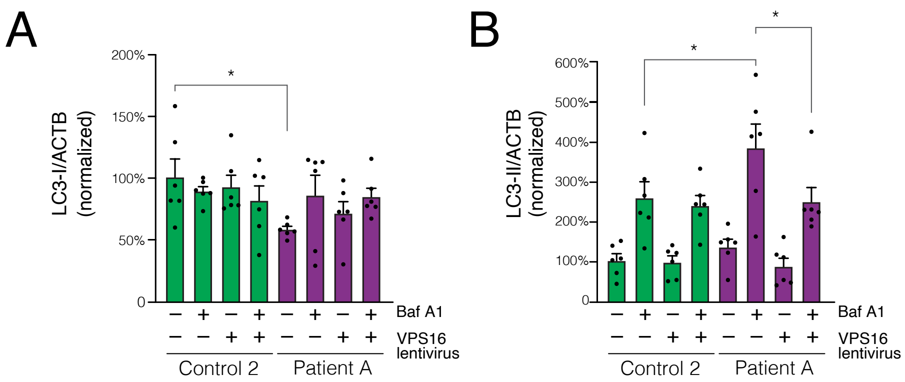

Appendix Figure S1 (related to Fig 6C-D). Quantifications of LC3-I (A) and LC3-II (B) normalized to β-actin (ACTB) and expressed as % of controls (n=6 biological replicates). Bar graphs represent data as mean ±SEM. Statistical comparisons between the indicated groups by one-way ANOVA with Holm-Sidak's multiple comparisons tests. * $p < 0.05$.

Appendix Table S1 – Summary of statistical tests with exact n- and p-values.

		n	comparison	Statistical test	p-value (adj)
Figure 3B	Ctrl 2	3	Ctrl 2 vs Pat A	2-tailed unpaired t-test	0.0184
VPS16 Exon 7-9	Pat A	3			
Figure 3B	Ctrl 2	3	Ctrl 2 vs Pat A	2-tailed unpaired t-test	0.0005
VPS16 Exon 22-24	Pat A	3			
Figure 3C	Ctrl 1	4	Ctrl 1 vs Pat A	one-way ANOVA w Dunnett's test	0.0155
	Ctrl 2	4	Ctrl 2 vs Pat A	one-way ANOVA w Dunnett's test	0.0075
	Pat A	4			
	Ctrl 2	3	Ctrl 2 vs Pat B	2-tailed unpaired t-test	<0.0001
	Pat B	3			
Figure 3E	Ctrl 1	4	Ctrl 1 vs Pat A	one-way ANOVA w Dunnett's test	0.0128
	Ctrl 2	4	Ctrl 2 vs Pat A	one-way ANOVA w Dunnett's test	0.0065
	Pat A	4			
	Ctrl 2	3	Ctrl 2 vs Pat B	2-tailed unpaired t-test	0.0003
	Pat B	3			
Figure 3F	Ctrl 2	3	Ctrl 2 vs Pat A	2-tailed unpaired t-test	0.0029
	Pat A	3			
	Ctrl 2	3	Ctrl 2 vs Pat B	2-tailed unpaired t-test	0.0002
	Pat B	3			
Figure 3G	Ctrl 2	3	Ctrl 2 vs Pat A	2-tailed unpaired t-test	0.0027
	Pat A	3			
Figure 3H	Ctrl 1	3	Ctrl 1 vs Pat A	one-way ANOVA w Dunnett's test	0.999
VPS33A	Ctrl 2	3	Ctrl 2 vs Pat A	one-way ANOVA w Dunnett's test	0.9727
	Pat A	3			
Figure 3H	Ctrl 1	3	Ctrl 1 vs Pat A	one-way ANOVA w Dunnett's test	0.3415
VPS11	Ctrl 2	3	Ctrl 2 vs Pat A	one-way ANOVA w Dunnett's test	0.6136
	Pat A	3			
Figure 3I	Ctrl	3	Ctrl vs VPS16	2-tailed unpaired t-test	<0.0001
VPS16	VPS16	3			
Figure 3I	Ctrl	3	Ctrl vs VPS16	2-tailed unpaired t-test	<0.0001
VPS33A	VPS16	3			
Figure 3I	Ctrl	3	Ctrl vs VPS16	2-tailed unpaired t-test	0.0141
VPS11	VPS16	3			
Figure 4B	Ctrl 2 + Ctrl	3	Ctrl 2 + Ctrl vs Pat A + Ctrl	one-way ANOVA w Holm-Sidak's test	0.9448
Puncta number	Ctrl 2 + VPS16	3	Ctrl 2 + Ctrl vs Ctrl 2 + VPS16	one-way ANOVA w Holm-Sidak's test	0.8471
	Pat A + Ctrl	3			
	Pat A + VPS16	3	Pat A + Ctrl vs Pat A + VPS16	one-way ANOVA w Holm-Sidak's test	>0.9999
Figure 4B	Ctrl 2 + Ctrl	3	Ctrl 2 + Ctrl vs Pat A + Ctrl	one-way ANOVA w Holm-Sidak's test	0.9111
Puncta intensity	Ctrl 2 + VPS16	3	Ctrl 2 + Ctrl vs Ctrl 2 + VPS16	one-way ANOVA w Holm-Sidak's test	0.9111
	Pat A + Ctrl	3			
	Pat A + VPS16	3	Pat A + Ctrl vs Pat A + VPS16	one-way ANOVA w Holm-Sidak's test	0.4749
Figure 4D	Ctrl 2 + Ctrl	29	Ctrl 2 + Ctrl vs Pat A + Ctrl	one-way ANOVA w Holm-Sidak's test	0.079
Puncta number	Ctrl 2 + VPS16	28			
	Ctrl 2 + VPS16[N52K]	30			
	Pat A + Ctrl	29	Pat A + Ctrl vs Pat A + VPS16	one-way ANOVA w Holm-Sidak's test	0.0086
	Pat A + VPS16	30	Pat A + Ctrl vs Pat A + VPS16[N52K]	one-way ANOVA w Holm-Sidak's test	<0.0001
	Pat A + VPS16[N52K]	30			
Figure 4D	Ctrl 2 + Ctrl	29	Ctrl 2 + Ctrl vs Pat A + Ctrl	one-way ANOVA w Holm-Sidak's test	<0.0001
Puncta intensity	Ctrl 2 + VPS16	28			
	Ctrl 2 + VPS16[N52K]	30			
	Pat A + Ctrl	29	Pat A + Ctrl vs Pat A + VPS16	one-way ANOVA w Holm-Sidak's test	0.0093
	Pat A + VPS16	30	Pat A + Ctrl vs Pat A + VPS16[N52K]	one-way ANOVA w Holm-Sidak's test	0.0093
	Pat A + VPS16[N52K]	30			
Figure 4F	Ctrl 2 + Ctrl	9	Ctrl 2 + Ctrl vs Pat A + Ctrl	one-way ANOVA w Holm-Sidak's test	0.0334
	Ctrl 2 + VPS16	9	Ctrl 2 + Ctrl vs Ctrl 2 + VPS16	one-way ANOVA w Holm-Sidak's test	0.799
	Pat A + Ctrl	9			
	Pat A + VPS16	9	Pat A + Ctrl vs Pat A + VPS16	one-way ANOVA w Holm-Sidak's test	0.0334
Figure 4G	Ctrl 2 + Ctrl	10	Ctrl 2 + Ctrl vs Pat A + Ctrl	one-way ANOVA w Holm-Sidak's test	0.0039
	Ctrl 2 + VPS16	10			
	Pat A + Ctrl	10	Pat A + Ctrl vs Pat A + VPS16	one-way ANOVA w Holm-Sidak's test	0.0248
	Pat A + VPS16	10			
Figure 4H	Ctrl 2 + Ctrl	10	Ctrl 2 + Ctrl vs Pat A + Ctrl	one-way ANOVA w Holm-Sidak's test	0.0037
	Ctrl 2 + VPS16	10			
	Pat A + Ctrl	10	Pat A + Ctrl vs Pat A + VPS16	one-way ANOVA w Holm-Sidak's test	0.0655
	Pat A + VPS16	10			
Figure 5B	Ctrl 2 + Ctrl	3	Ctrl 2 + Ctrl vs Pat A + Ctrl	one-way ANOVA w Holm-Sidak's test	0.0159
Puncta number	Ctrl 2 + VPS16	3	Ctrl 2 + Ctrl vs Ctrl 2 + VPS16	one-way ANOVA w Holm-Sidak's test	0.809
	Pat A + Ctrl	3			
	Pat A + VPS16	3	Pat A + Ctrl vs Pat A + VPS16	one-way ANOVA w Holm-Sidak's test	0.0159
Figure 5B	Ctrl 2 + Ctrl	3	Ctrl 2 + Ctrl vs Pat A + Ctrl	one-way ANOVA w Holm-Sidak's test	0.0019
Puncta intensity	Ctrl 2 + VPS16	3	Ctrl 2 + Ctrl vs Ctrl 2 + VPS16	one-way ANOVA w Holm-Sidak's test	0.5012
	Pat A + Ctrl	3			
	Pat A + VPS16	3	Pat A + Ctrl vs Pat A + VPS16	one-way ANOVA w Holm-Sidak's test	0.0015

Figure 5C	Ctrl 2<br>Pat A	3<br>3		*no statistical comparison was done*	
Figure 5D	Ctrl 2<br>Pat A	3<br>3		*no statistical comparison was done*	
Figure 5E	Ctrl 2<br>Pat A	3<br>3	Ctrl 2 vs Pat A	2-tailed unpaired t-test	0.0677
Figure 6B	Ctrl 2 + Ctrl	3	Ctrl 2 + Ctrl vs Pat A + Ctrl	one-way ANOVA w Holm-Sidak's test	0.6905
	Ctrl 2 + Ctrl, starv	3	Ctrl 2 + Ctrl, starv vs Pat A + Ctrl, starv	one-way ANOVA w Holm-Sidak's test	0.1538
	Ctrl 2 + VPS16	3	Ctrl 2 + Ctrl vs Ctrl 2 + Ctrl, starv	one-way ANOVA w Holm-Sidak's test	0.6905
	Ctrl 2 + VPS16, starv	3			
	Pat A + Ctrl	3	Pat A + Ctrl vs Pat A + Ctrl, starv	one-way ANOVA w Holm-Sidak's test	0.6905
	Pat A + Ctrl, starv	3			
	Pat A + VPS16	3	Pat A + Ctrl vs Pat A + VPS16	one-way ANOVA w Holm-Sidak's test	0.4101
	Pat A + VPS16, starv	3	Pat A + Ctrl, starv vs Pat A + VPS16, starv	one-way ANOVA w Holm-Sidak's test	0.3498
Figure 6D	Ctrl 2 + Ctrl	6	Ctrl 2 + Ctrl vs Pat A + Ctrl	one-way ANOVA w Holm-Sidak's test	0.0149
	Ctrl 2 + Ctrl + Baf A1	6	Ctrl 2 + Ctrl + Baf A1 vs Pat A + Ctrl + Baf A1	one-way ANOVA w Holm-Sidak's test	0.0512
	Ctrl 2 + VPS16	6			
	Ctrl 2 + VPS16 + Baf A1	6			
	Pat A + Ctrl	6	Pat A + Ctrl vs Pat A + VPS16	one-way ANOVA w Holm-Sidak's test	0.0002
	Pat A + Ctrl + Baf A1	6	Pat A + Ctrl + Baf A1 vs Pat A + VPS16 + Baf A1	one-way ANOVA w Holm-Sidak's test	0.0004
	Pat A + VPS16	6			
	Pat A + VPS16 + Baf A1	6			
Figure 6E	Ctrl 2 + Ctrl	6	Ctrl 2 + Ctrl vs Pat A + Ctrl	one-way ANOVA w Holm-Sidak's test	0.9473
	Ctrl 2 + VPS16	6	Ctrl 2 + VPS16 vs Pat A + VPS16	one-way ANOVA w Holm-Sidak's test	0.7201
	Pat A + Ctrl	6	Pat A + Ctrl vs Pat A + VPS16	one-way ANOVA w Holm-Sidak's test	0.7201
	Pat A + VPS16	6			
Figure 6G	Ctrl 2	78	Ctrl 2 vs Pat A	one-way ANOVA w Holm-Sidak's test	<0.0001
	Ctrl 2, starv	61	Ctrl 2 vs Ctrl 2, starv	one-way ANOVA w Holm-Sidak's test	0.0339
	Pat A	113	Pat A vs Pat A, starv	one-way ANOVA w Holm-Sidak's test	0.0405
	Pat A, starv	57			
Figure 6H	Ctrl 2	78	Ctrl 2 vs Pat A	one-way ANOVA w Holm-Sidak's test	<0.0001
	Ctrl 2, starv	59	Ctrl 2 vs Ctrl 2, starv	one-way ANOVA w Holm-Sidak's test	0.1502
	Pat A	113	Pat A vs Pat A, starv	one-way ANOVA w Holm-Sidak's test	0.0145
	Pat A, starv	50	Ctrl 2, starv vs Pat A, starv	one-way ANOVA w Holm-Sidak's test	<0.0001
Figure 7D	Ctrl<br>vps16	6<br>11	Ctrl vs vps16	Mann-Whitney U test	0.0019
Figure 8B	Ctrl, 3dpf<br>vps16, 3dpf	6<br>6	Ctrl, 3dpf vs vps16, 3dpf	Mann-Whitney U test	0.3939
	Ctrl, 5 dpf<br>vps16, 5 dpf	3<br>12	Ctrl, 5 dpf vs vps16, 5 dpf	Mann-Whitney U test	0.0044
Figure 8E	Ctrl<br>vps16	28<br>14	Ctrl vs vps16	Mann-Whitney U test	0.0001
Figure EV3B<br>VPS16	Ctrl 2; 0 min<br>Pat A; 0 min<br>Ctrl 2; 30 min<br>Pat A; 30 min<br>Ctrl 2; 120 min<br>Pat A; 120 min	3<br>3<br>3<br>3<br>3<br>3		*no statistical comparison was done*	
Figure EV3B<br>VPS33A	Ctrl 2; 0 min<br>Pat A; 0 min<br>Ctrl 2; 30 min<br>Pat A; 30 min<br>Ctrl 2; 120 min<br>Pat A; 120 min	2<br>2<br>2<br>2<br>2<br>2		*no statistical comparison was done*	
Figure EV3D<br>VPS16	Ctrl<br>VPS16	3<br>3	Ctrl vs VPS16	2-tailed unpaired t-test	<0.0001
Figure EV3D<br>VPS33A	Ctrl<br>VPS16	3<br>3	Ctrl vs VPS16	2-tailed unpaired t-test	<0.0001
Figure EV4A	Ctrl 2<br>Pat A	3<br>3	Ctrl 2 vs Pat A	2-tailed unpaired t-test	0.0023
Figure EV4B	Ctrl 2 + Ctrl<br>Ctrl 2 + Ctrl, starv<br>Ctrl 2 + VPS16<br>Ctrl 2 + VPS16, starv<br>Pat A + Ctrl<br>Pat A + Ctrl, starv<br>Pat A + VPS16<br>Pat A + VPS16, starv	3<br>3<br>3<br>3<br>3<br>3<br>3<br>3		*no statistical comparison was done*	
Figure EV4D	Ctrl 2<br>Pat A	10<br>10		*no statistical comparison was done*	
Figure EV4E	Ctrl 2 + Ctrl	8	Ctrl 2 + Ctrl vs Pat A + Ctrl	one-way ANOVA w Holm-Sidak's test	0.4238
Puncta number	Ctrl 2 + VPS16	7	Ctrl 2 + Ctrl vs Ctrl 2 + VPS16	one-way ANOVA w Holm-Sidak's test	0.1783
	Pat A + Ctrl	8			
	Pat A + VPS16	6	Pat A + Ctrl vs Pat A + VPS16	one-way ANOVA w Holm-Sidak's test	0.7075

Figure EV4E	Ctrl 2 + Ctrl	8		*no statistical comparison was done*	
Puncta intensity	Ctrl 2 + VPS16	7			
	Pat A + Ctrl	8			
	Pat A + VPS16	6			
Appendix Figure S1A	Ctrl 2 + Ctrl	6	Ctrl 2 + Ctrl vs Pat A + Ctrl	one-way ANOVA w Holm-Sidak's test	0.0262
	Ctrl 2 + Ctrl + Baf A1	6	Ctrl 2 + Ctrl + Baf A1 vs Pat A + Ctrl + Baf A1	one-way ANOVA w Holm-Sidak's test	0.9692
	Ctrl 2 + VPS16	6			
	Ctrl 2 + VPS16 + Baf A1	6			
	Pat A + Ctrl	6	Pat A + Ctrl vs Pat A + VPS16	one-way ANOVA w Holm-Sidak's test	0.7683
	Pat A + Ctrl + Baf A1	6	Pat A + Ctrl + Baf A1 vs Pat A + VPS16 + Baf A1	one-way ANOVA w Holm-Sidak's test	0.9692
	Pat A + VPS16	6			
	Pat A + VPS16 + Baf A1	6			
Appendix Figure S1B	Ctrl 2 + Ctrl	6	Ctrl 2 + Ctrl vs Pat A + Ctrl	one-way ANOVA w Holm-Sidak's test	0.502
	Ctrl 2 + Ctrl + Baf A1	6	Ctrl 2 + Ctrl + Baf A1 vs Pat A + Ctrl + Baf A1	one-way ANOVA w Holm-Sidak's test	0.0258
	Ctrl 2 + VPS16	6			
	Ctrl 2 + VPS16 + Baf A1	6			
	Pat A + Ctrl	6	Pat A + Ctrl vs Pat A + VPS16	one-way ANOVA w Holm-Sidak's test	0.502
	Pat A + Ctrl + Baf A1	6	Pat A + Ctrl + Baf A1 vs Pat A + VPS16 + Baf A1	one-way ANOVA w Holm-Sidak's test	0.0197
	Pat A + VPS16	6			
	Pat A + VPS16 + Baf A1	6			
