# Supplementary material for: Bi‐allelic VPS16 variants limit HOPS/CORVET levels and cause a mucopolysaccharidosis‐like disease
Source: EMBO Mol Med. 2021 May 3;13(5):e13376. doi: 10.15252/emmm.202013376 (PMC8103096; doi:10.15252/emmm.202013376)
Supplement: Supplementary file 2 — Expanded View Figures PDF [file EMMM-13-e13376-s002.pdf]

Expanded View Figures

A

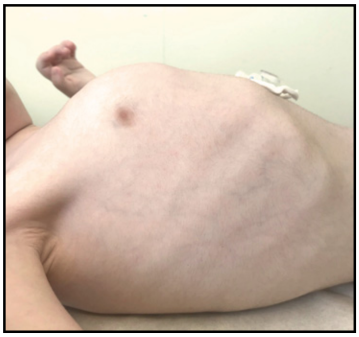

B

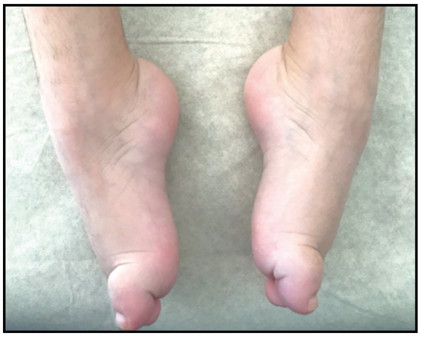

C

Age	2.5 yrs	3.8 yrs	(Reference)
Hemoglobin	11.2 g/dL (10.5–13.5)	10.8 g/dL (11.5–13.5)	
MCV		84 fL	(75–87 fL)
MCH		28 pg	(24–30 pg)
TIBC	55 μmol/L	59 μmol/L	(48–83 μmol/L)
Ferritin	72 μg/L	52 μg/L	(20–200)
Iron	11 μmol/L	8 μmol/L	(9–34 μmol/L)
Transferrin		2.37 g/L	(1.94–3.26 g/L)
Transferrin Sat	21%	13%	(9–50%)
Transferrin Rec	9.2 mg/L	10.8 mg/L	(2.2–5.0)

Figure EV1. Additional clinical features of patient A.

A, B Photographs at 3.5 years of age. (A) Barrel-shaped chest with pectus carinatum. (B) Pes cavus with hammertoe deformity of first toes bilaterally.

C Blood and plasma iron status at indicated ages. The patient received oral iron supplementation. Values outside reference range are colored red. MCV, mean corpuscular volume; MCH, mean corpuscular hemoglobin; TIBC, total iron-binding capacity.

© 2021 The Authors

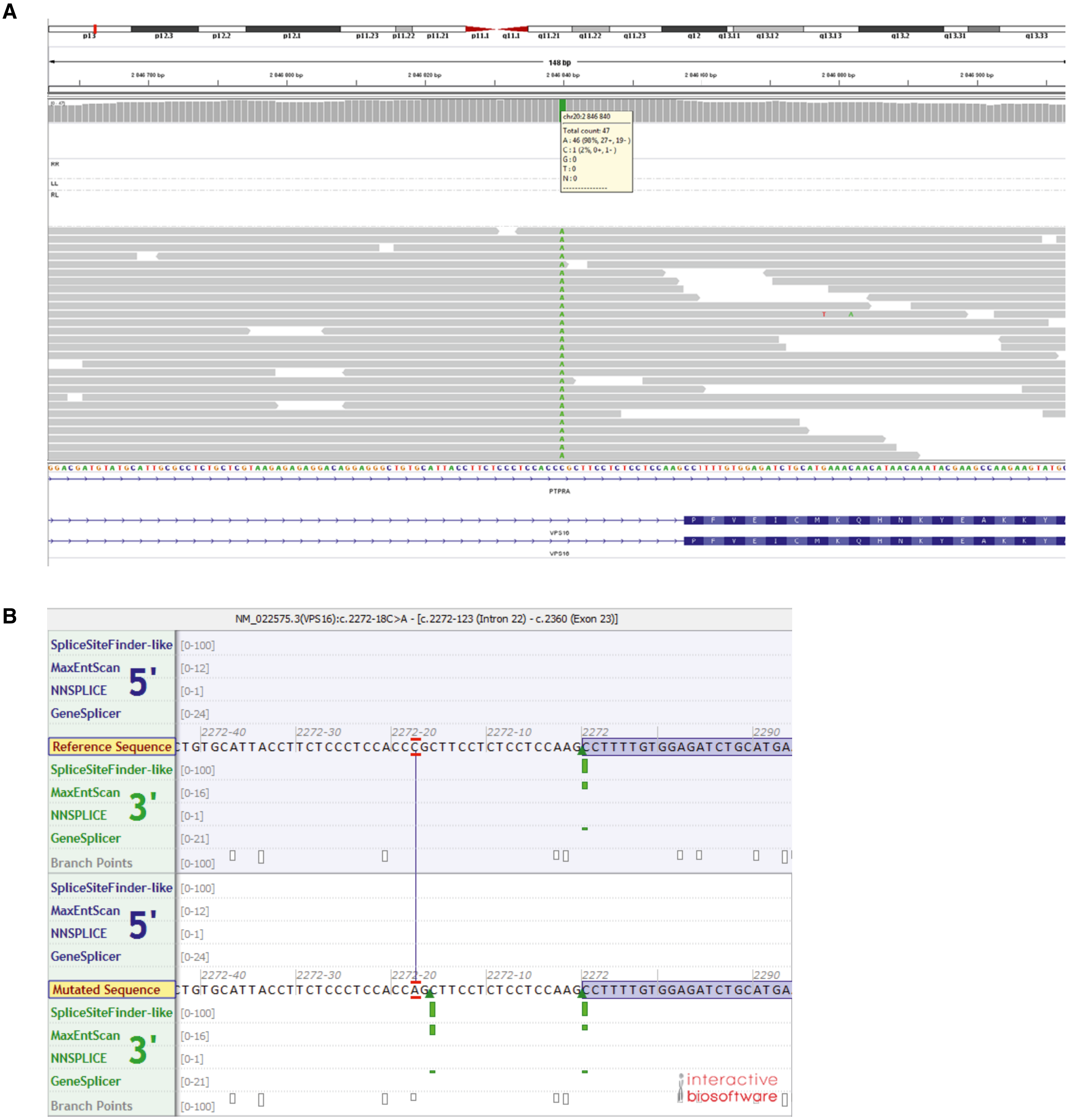

Figure EV2. Whole-genome sequencing and mutation prediction.

A Assembly of whole-genome sequencing reads, identifying the VPS16 c.2272-18 variant (in patient A).

B Predicted impact by the c.2272-18C>A variant on splicing, as predicted by algorithms used by Alamut Visual (Interactive Biosoftware). Green bars indicate predicted splice-acceptor sites.

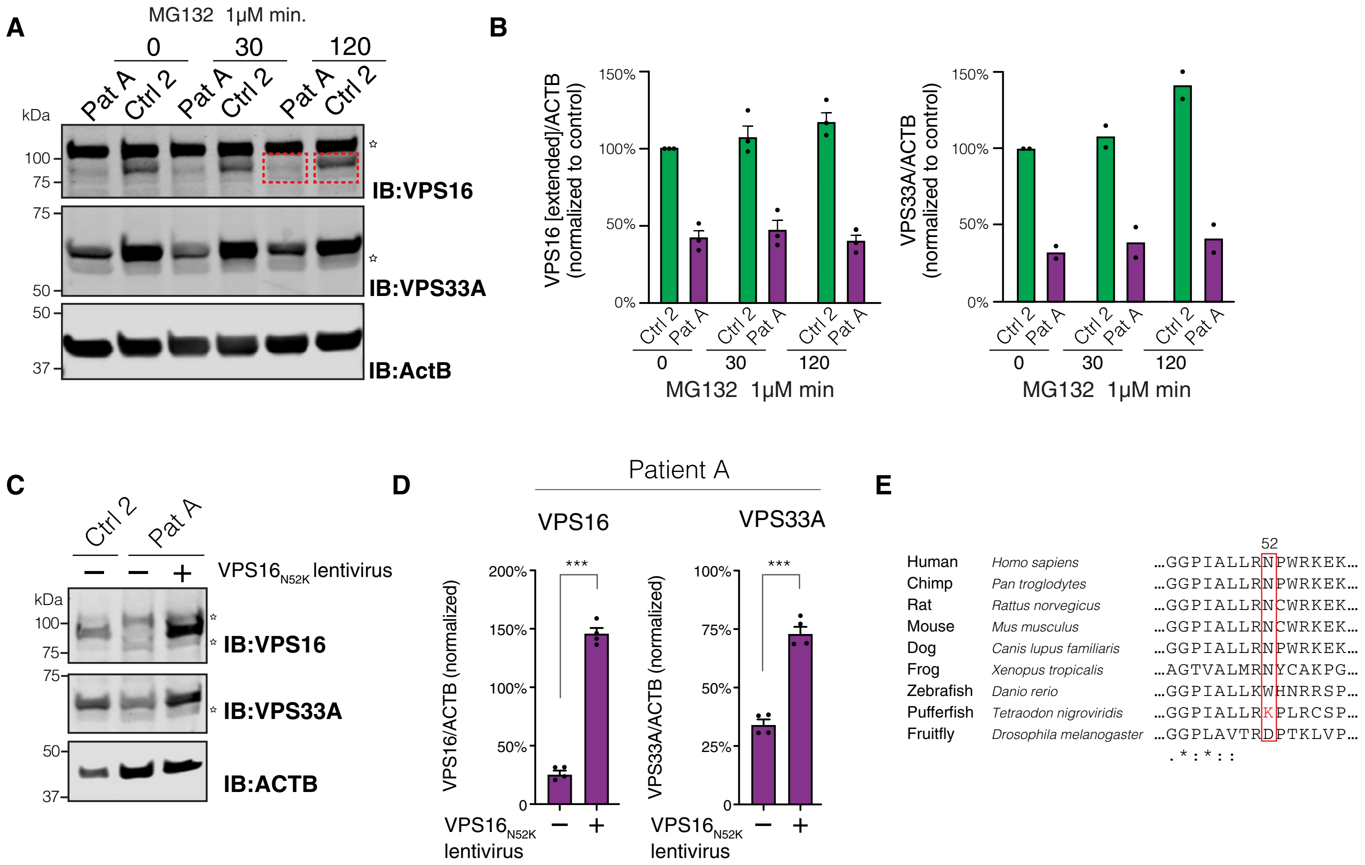

Figure EV3. Analysis of HOPS/CORVET subunits in fibroblasts (related to Fig 3).

A, B Effect of proteasome inhibition on VPS16 and VPS33A. (A) Immunoblots for VPS16 and VPS33A and quantifications of protein levels in lysates of fibroblasts treated with MG132 for 30 or 120 min, respectively. VPS16 band densities were quantified in an extended area (indicated by red boxes) to capture the presence of a possible truncated protein with a molecular weight of 87 kDa (wild-type VPS16 is 95 kDa). Stars denote unspecific bands. (B) Quantifications of (A) normalized to control conditions and represented as mean ± SEM for VPS16 (n = 3 biological replicates) or mean for VPS33A (n = 2 biological replicates).

C–E Rescue of cellular phenotype by VPS^{N52K}. (C) Representative immunoblots of fibroblast lysates using antibodies against VPS16, VPS33A, and actin. Stars denote unspecific bands. (D) Levels of VPS16 and VPS33A quantified in patient cells transduced with control or VPS16^{N52K}-expressing lentiviruses and normalized to levels of actin (n = 3). Data represented as mean ± SEM; ***P < 0.001 by unpaired Student's t-tests. (E) Cross-species sequence alignment of VPS16 residues surrounding the asparagine in position 52; (bottom row) asterisks, colons, and periods indicate residues that are fully, strongly, or weakly conserved, respectively.

 © 2021 The Authors

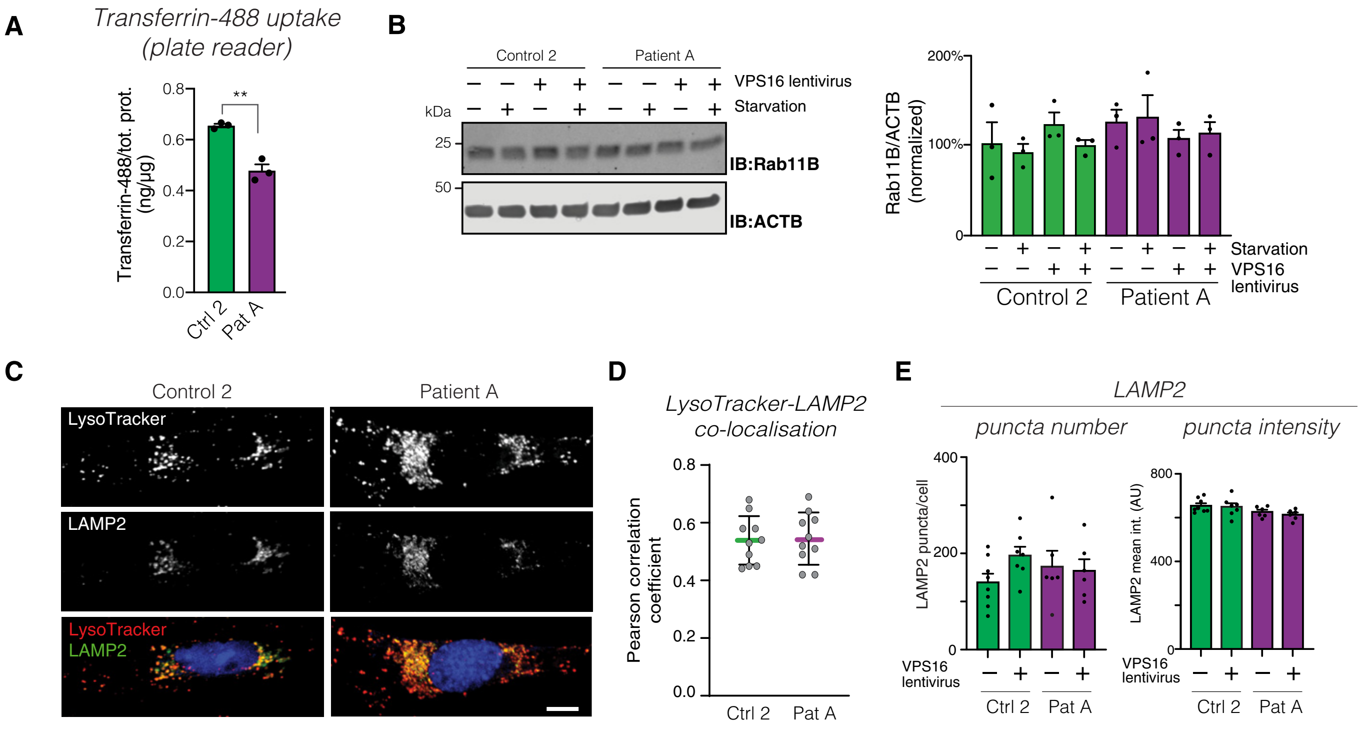

Figure EV4. Analysis of endolysosomal compartments (related to Figs 4 and 5).

A Quantification of cellular uptake of fluorescently labeled transferrin in fibroblast lysates, normalized to total protein levels (n = 3 biological replicates). Data represented as mean ±SEM. Statistical comparisons by unpaired two-tailed Student's t-test (n = 3; P = 0.0023). **P < 0.01.

B Immunoblot (left) of RAB11B in the indicated fibroblasts, under basal and serum-starved conditions, and (right) summary quantifications normalized to levels of actin (ACTB) and expressed as % of controls. Bar graphs represent data as mean ±SEM (n = 3 biological replicates).

C Representative confocal micrographs of fibroblasts stained for LAMP2 and LysoTracker. Scale bar 10 µm.

D Quantification of the co-localization between LysoTracker and LAMP2, expressed as Pearson correlation coefficients. Colored horizontal bars indicate the median values and whiskers 5 and 95 percentiles (n = 10 optical sections from three independent experiments).

E Quantification of the number and intensities of LAMP2-stained puncta. Data represented as mean ± SEM (n = 6).

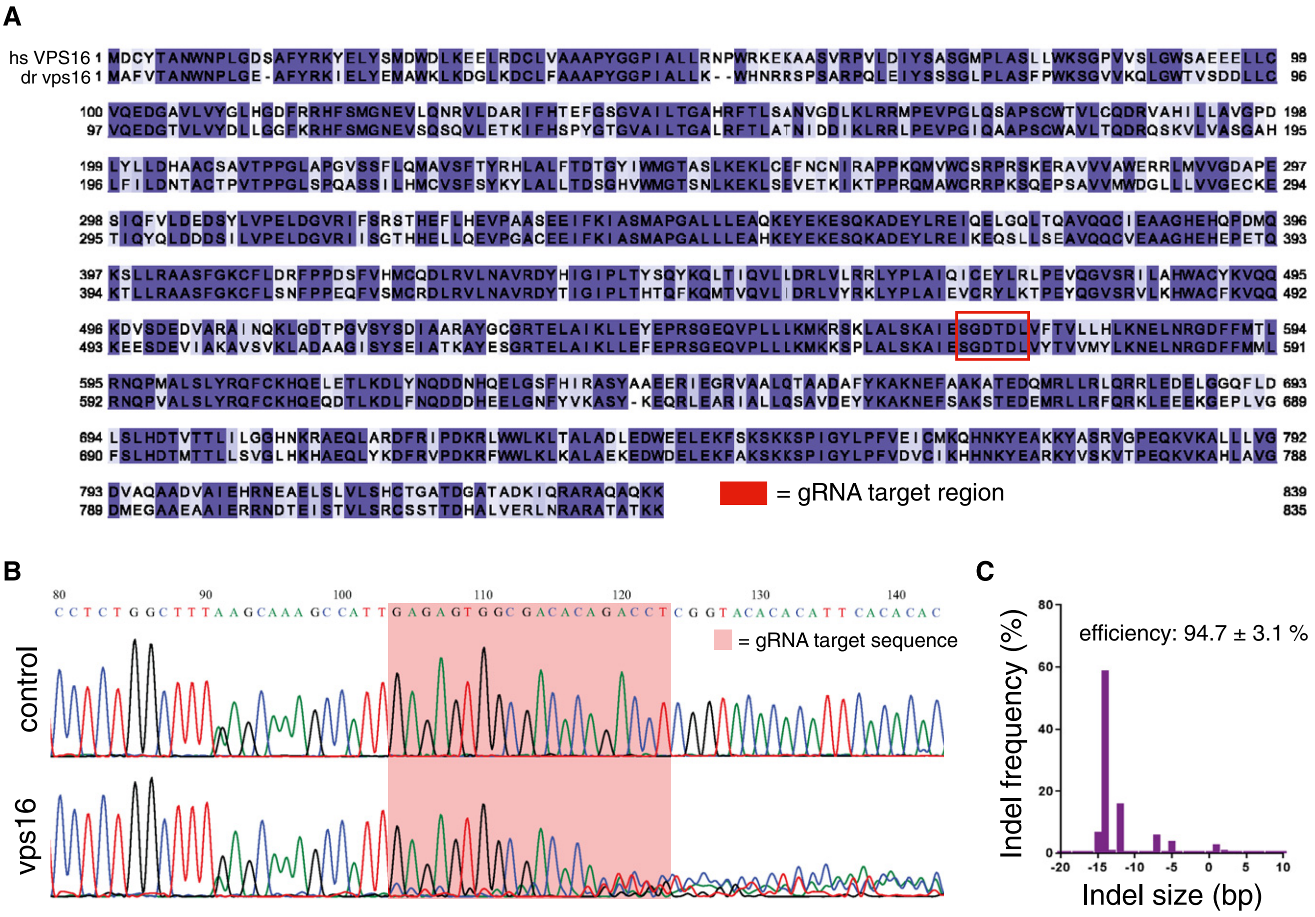

Figure EV5. Sequence validation of the *vps16* crispant zebrafish embryos.

A Amino acid sequence alignment of the human and zebrafish VPS16 proteins, colored according to homology and with the region of the gRNA target site indicated.
B Representative sequence trace for a zebrafish *vps16* embryo as compared to a control, showing significant decomposition. The gRNA target sequence is highlighted by the shaded box.
C Representative indel distribution for a sequenced *vps16* embryo.

 © 2021 The Authors
