# Supplementary material for: Bi‐allelic VPS16 variants limit HOPS/CORVET levels and cause a mucopolysaccharidosis‐like disease
Source: EMBO Mol Med. 2021 May 3;13(5):e13376. doi: 10.15252/emmm.202013376 (PMC8103096; doi:10.15252/emmm.202013376)
Supplement: Supplementary file 3 — Table EV1 [file EMMM-13-e13376-s005.docx]

Table EV1 – Genes analyzed in the gene panels used to investigate patients.

|  | Gene panel | Subject | Genes included |
| --- | --- | --- | --- |
| A) | LSD gene panel | Patient A | *AGA, AP3B1, ARSA, ARSB, ASAH1, ATP13A2, BLOC1S3, CLN3, CLN5, CLN6, CLN8, CTNS, CTSA, CTSD, CTSF, CTSK, DNAJC5, DTNBP1, FUCA1, GAA, GALC, GALNS, GBA, GLA, GLB1, GM2A, GNE, GNPTAB, GNPTG, GRN, GNS, GUSB, HEXA, HEXB, HGSNAT, HPS1, HPS3, HPS4, HPS5, HPS6, HYAL1, IDS, IDUA, KCTD7, LAMP2, LIPA, MAN2B1, MANBA, MCOLN1, MFSD8, NAGA, NAGLU, NEU1, NPC1, NPC2, PPT1, PSAP, SCARB2, SGSH, SLC17A5, SMPD1, SUMF1, TPP1, ABCD1, ABCD3, ACBD5, ACOX1, AGPS, AGXT, AMACR, BAAT, CAT, DNM1L, FAR1, GNPAT, HSD17B4, MFF, MVK, PEX1, PEX10, PEX11B, PEX12, PEX13, PEX14, PEX16, PEX19, PEX2, PEX26, PEX3, PEX5, PEX6, PEX7, PHYH, SCP2, TRIM37, DHCR7, CYP27A1* |
| B) | Leukodystrophy gene panel | Patient B | *BTD, EIF2B1, EIF2B2, EIF2B3, EIF2B4, EIF2B5, FOLR1, GBA, PNPO, SCARB2, SCN2A* |
| C) | Lysoplex *in silico* gene panel (Di Fruscio et al. 2015) | Patient A Patient B | *AAGAB, AAK1, ABCA1, ABCA2, ABCA5, ABCA7, ABCB9, ABL1, ABL2, ACAP1, ACAP2, ACAP3, ACP2, ACP5, ADA, ADAM8, ADAMTSL2, ADAP1, ADRB1, ADRB2, ADRB3, ADRBK1, ADRBK2, AGA, AGAP1, AGAP2, AGFG1, AHSG, AMBRA1, AMPH, ANO1, AP1AR, AP1B1, AP1G1, AP1G2, AP1M1, AP1M2, AP1S1, AP1S2, AP1S3, AP2A1, AP2A2, AP2B1, AP2M1, AP2S1, AP3B1, AP3B2, AP3D1, AP3M1, AP3M2, AP3S1, AP3S2, AP4B1, AP4E1, AP4M1, AP4S1, AP5B1, AP5M1, AP5S1, AP5Z1, APOA1, APOE, APP, APPL1, ARAP1, ARAP2, ARAP3, ARF1, ARF3, ARF4, ARF5, ARF6, ARFGAP1, ARFGAP2, ARFGAP3, ARFGEF1, ARFGEF2, ARFIP1, ARFIP2, ARFRP1, ARHGAP27, ARHGAP33, ARL1, ARL10, ARL11, ARL13A, ARL13B, ARL14, ARL14EP, ARL14EPL, ARL15, ARL16, ARL17A, ARL17B, ARL2, ARL2BP, ARL3, ARL4A, ARL4C, ARL4D, ARL5A, ARL5B, ARL5C, ARL6, ARL6IP1, ARL6IP4, ARL6IP5, ARL6IP6, ARL8A, ARL8B, ARL9, ARRB1, ARRB2, ARSA, ARSB, ARSD, ARSG, ASAH1, ASAP1, ASAP2, ASAP3, ASGR1, ATG10, ATG12, ATG13, ATG14, ATG16L1, ATG16L2, ATG2A, ATG2B, ATG3, ATG4A, ATG4B, ATG4C, ATG4D, ATG5, ATG7, ATG9A, ATG9B, ATP13A2, ATP6AP1, ATP6AP1L, ATP6AP2, ATP6V0A1, ATP6V0A2, ATP6V0A4, ATP6V0B, ATP6V0C, ATP6V0D1, ATP6V0D2, ATP6V0E1, ATP6V0E2, ATP6V1A, ATP6V1B1, ATP6V1B2, ATP6V1C1, ATP6V1C2, ATP6V1D, ATP6V1E1, ATP6V1E2, ATP6V1F, ATP6V1G1, ATP6V1G2, ATP6V1G3, ATP6V1H, AZU1, BCL10, BECN1, BIN1, BLOC1S1, BLOC1S2, BLOC1S3, BLOC1S4, BLOC1S5, BLOC1S6, BNIP1, BST1, c12orf44, C1orf85, C2orf18, CAV1, CAV2, CAV3, CBL, CBLB, CBLC, CCR5, CCZ1, CD1B, CD1D, CD1E, CD207, CD2AP, CD63, CD68, CDC42, CDH13, CFTR, CHID1, CHIT1, CHM, CHML, CHMP1B, CHMP2A, CHMP2B, CHMP3, CHMP4A, CHMP4B, CHMP4C, CHMP5, CHMP6, CHPT1, CISD2, CLCN1, CLCN2, CLCN3, CLCN4, CLCN5, CLCN6, CLCN7, CLCNKA, CLCNKB, CLINT1, CLN3, CLN5, CLN6, CLN8, CLTA, CLTB, CLTC, CLTCL1, CLU, CPVL, CR1, CREG1, CSF1R, CST3, CTBS, CTNS, CTSA, CTSB, CTSC, CTSD, CTSE, CTSF, CTSH, CTSK, CTSL1, CTSL2, CTSO, CTSS, CTSZ, CTTN, CUBN, CXCR1, CXCR2, CXCR4, CYTH2, DAB2, DAP, DAPK1, DAPK2, DAPK3, DCTN1, DMBT1, DNAJC5, DNAJC6, DNASE2, DNM1, DNM1L, DNM2, DNM3, DOC2A, DRAM1, DRAM2, DTNBP1, DYM, DYNC1H1, ECM1, EEA1, EGF, EGFR, EHD1, EHD2, EHD3, EHD4, EI24, EIF4G1, ENTPD4, EPDR1, EPG5, EPN1, EPN2, EPN3, EPS15, EPS15L1, ERBB2, ERBB3, ERBB4, ERRFI1, F2R, FAM125A, FAM125B, FAM176A, FAM48A, FAN1, FBN1, FBXO7, FGD1, FGD2, FGD4, FGFR2, FGFR3, FGFR4, FIG4, FIS1, FLT1, FNBP1, FOLR1, FOXO1, FOXO3, FOXO4, FOXO6, FUCA1, FUNDC1, FYCO1, GAA, GABARAP, GABARAPL2, GALC, GALNS, GATA2, GBA, GGA1, GGA2, GGA3, GGH, GIT1, GIT2, GLA, GLB1, GM2A, GNE, GNPTAB, GNPTAG, GNS, GOLIM4, GPR137B, GRK1, GRK4, GRK5, GRK6, GRK7, GRN, GUSB, HCK, HDAC6, HERC1, HEXA, HEXB, HFE, HGS, HGSNAT, HIP1, HIP1R, HLA-A, HLA-B, HLA-C, HLA-E, HLA-F, HLA-G, HPS1, HPS4, HPSE, HRAS, HSPA1A, HSPA1B, HSPA1L, HSPA2, HSPA6, HSPA8, HTR2B, HYAL1, HYAL2, HYAL3, IDS, IDUA, IFI30, IFT27, IGF1R, IGF2R, IL2RA, IL2RB, IL2RG, INPP1, INPP4A, INPP4B, INPP5A, INPP5B, INPP5D, INPP5E, INPP5F, INPP5J, INPP5K, INPPL1, IQSEC1, IQSEC2, IQSEC3, IRGM, ITCH, ITM2C, ITSN1, ITSN2, KCNE1, KCNE2, KCTD7, KDR, KIAA0226, KIAA1324, KIF16B, KIT, KRT18, KRT8, LAMP1, LAMP2, LAMP3, LAPTM4A, LAPTM4B, LAPTM5, LDLR, LDLRAP1, LGMN, LIPA, LITAF, LMBRD1, LPL, LRBA, LRP1B, LRP2, LRP3, LRRK2, LYST, M6PR, MAN2B1, MAN2B2, MANBA, MAP1LC3A, MAP1LC3B, MAP1LC3B2, MAP1LC3C, MAPT, MARCH8, MCOLN1, MCOLN2, MCOLN3, MDM2, MET, MFSD1, MFSD8, MITF, MLPH, MPO, MRC1, MSR1, MT3, MTDH, MTM1, MTMR1, MTMR10, MTMR11, MTMR12, MTMR14, MTMR2, MTMR3, MTMR4, MTMR6, MTMR7, MTMR8, MTMR9, MTOR, MTSS1, MYO1C, MYO5A, MYO5B, MYO6, MYO7A, NAAA, NAGA, NAGLU, NAGPA, NBR1, NCF2, NCF4, NCSTN, NECAP1, NECAP2, NEDD4, NEDD4L, NEU1, NEU4, NPC1, NPC2, NTRK1, NUMB, NUMBL, OCRL, OSBP, OSBP2, OSBPL10, OSBPL11, OSBPL1A, OSBPL2, OSBPL3, OSBPL5, OSBPL6, OSBPL7, OSBPL8, OSBPL9, OSTM1, PA2G4, PACSIN3, PARD3, PARD6A, PARD6B, PARD6G, PARK2, PARK7, PCSK9, PCYOX1, PDCD6IP, PDGFRA, PDLIM7, PEBP4, PGCP, PI4K2A, PI4K2B, PI4KA, PI4KB, PICALM, PIK3C2A, PIK3C2B, PIK3C2G, PIK3C3, PIK3CA, PIK3CB, PIK3CD, PIK3CG, PIK3R1, PIK3R2, PIKFYVE, PIM2, PINK1, PIP4K2A, PIP4K2B, PIP4K2C, PIP5K1A, PIP5K1B, PIP5K1C, PIPSL, PITPNA, PITPNB, PITPNC1, PITPNM1, PITPNM3, PLA2G6, PLBD2, PLCB1, PLCG1, PLCG2, PLD1, PLD2, PLD3, PLEKHA8, PPT1, PPT2, PRCP, PRDX6, PRKAA1, PRKCI, PRKCZ, PSAP, PSD, PSD2, PSD3, PSD4, PSEN1, PSEN2, PTEN, RAB10, RAB11A, RAB11B, RAB11FIP1, RAB11FIP2, RAB11FIP3, RAB11FIP4, RAB11FIP5, RAB12, RAB13, RAB14, RAB15, RAB17, RAB18, RAB19, RAB1A, RAB1B, RAB20, RAB21, RAB22A, RAB23, RAB24, RAB25, RAB26, RAB27A, RAB27B, RAB28, RAB2A, RAB2B, RAB30, RAB31, RAB32, RAB33A, RAB33B, RAB34, RAB35, RAB36, RAB37, RAB38, RAB39A, RAB39B, RAB3A, RAB3B, RAB3C, RAB3D, RAB3IL1, RAB3IP, RAB40A, RAB40AL, RAB40B, RAB40C, RAB41, RAB42, RAB43, RAB44, RAB4A, RAB4B, RAB5A, RAB5B, RAB5C, RAB6A, RAB6B, RAB6C, RAB7A, RAB7L1, RAB8A, RAB8B, RAB9A, RAB9B, RABAC1, RABEP1, RABEP2, RABEPK, RABGAP1L, RABGEF1, RABIF, RABL2A, RABL2B, RABL3, RABL6, RAC1, RAMP2, RAMP3, RASEF, RB1CC1, RET, RGS19, RILP, RILPL1, RILPL2, RIMS1, RIMS2, RIN1, RIN2, RIN3, RNASET2, RNF13, RNF185, RNF41, RPH3A, RPS6KA3, RPTOR, RRAGA, RRAGB, RRAGC, RRAGD, RUFY1, SACM1L, SBF1, SBF2, SCAMP2, SCARB2, SCARF1, SCP2, SCPEP1, SEC14L3, SEC14L4, SERINC2, SFTPD, SGIP1, SGSH, SH3GL1, SH3GL2, SH3GL3, SH3GLB1, SH3GLB2, SH3KBP1, SH3TC2, SIAE, SIDT2, SIL1, SIRT1, SLC11A2, SLC15A3, SLC17A5, SLC26A11, SLC29A3, SLC36A1, SLC37A3, SMAP1, SMAP2, SMPD1, SMURF1, SMURF2, SNAP25, SNAP29, SNAP91, SNAPIN, SNCA, SNF8, SNX1, SNX10, SNX11, SNX12, SNX13, SNX14, SNX15, SNX16, SNX17, SNX18, SNX19, SNX2, SNX20, SNX21, SNX22, SNX24, SNX25, SNX27, SNX29, SNX3, SNX30, SNX31, SNX32, SNX33, SNX4, SNX5, SNX6, SNX7, SNX8, SNX9, SORL1, SPACA3, SPATA18, SPG20, SPHK2, SQSTM1, SRC, SRGN, STAB1, STAB2, STAM, STAM2, STAMBP, STARD3, STEAP3, STK11, STON1, STON2, STS, STX10, STX11, STX12, STX16, STX17, STX18, STX19, STX1A, STX1B, STX2, STX3, STX4, STX5, STX6, STX7, STX8, STXBP1, STXBP2, STXBP3, STXBP4, STXBP5, STXBP5L, STXBP6, SUMF1, SYNJ1, SYNJ2, SYNRG, SYP, SYPL1, SYPL2, SYT1, SYTL2, TCIRG1, TECPR1, TF, TFAP2A, TFE3, TFEB, TFEC, TFRC, TLR7, TLR9, TM9SF1, TMEM127, TMEM135, TMEM192, TMEM2, TMEM237, TMEM55A, TMEM55B, TMEM59, TMEM74, TMEM8A, TMEM9, TMEM92, TOM1, TPCN1, TPCN2, TPP1, TRAF6, TRIM13, TRIM23, TSG101, TSPAN1, TYR, TYRP1, ULK1, ULK2, ULK3, UNC93B1, USP8, UVRAG, VAMP4, VAMP8, VMA21, VMP1, VPS11, VPS16, VPS18, VPS25, VPS28, VPS35, VPS36, VPS37A, VPS37B, VPS37C, VPS37D, VPS39, VPS45, VPS4A, VPS4B, VTA1, WASL, WDR45, WDR45L, WDR48, WIPI1, WIPI2, WWP1, ZFYVE16, ZFYVE20, ZFYVE9, ZNRF1, ZNRF2* |
