# Supplementary material for: Bi‐allelic VPS16 variants limit HOPS/CORVET levels and cause a mucopolysaccharidosis‐like disease
Source: EMBO Mol Med. 2021 May 3;13(5):e13376. doi: 10.15252/emmm.202013376 (PMC8103096; doi:10.15252/emmm.202013376)
Supplement: Supplementary file 4 — Movie EV1 [file EMMM-13-e13376-s006.zip › Sofou_et_al_-_Legend_Movie_EV1.docx]

**Movie EV1. Phagocyting microglia in control fish.** Timelapse video of the optic tectum of a 5 dpf control *Tg(CMV:EGFP-map1lc3b)* embryo stained with LysoTracker. Microglia, identified by location, morphology, and LysoTracker phenotype, are seen performing phagocytosis. Video represents a 3 hour timeframe with images collected 6 min apart.
